# (1*H*-Benzimidazol-2-yl)methanaminium perchlorate–18-crown-6–water (1/1/1)

**DOI:** 10.1107/S1600536811050665

**Published:** 2011-12-03

**Authors:** Su-Wen Sun

**Affiliations:** a Ordered Matter Science Research Cente, College of Chemistry and Chemical Engineering, Southeast University, Nanjing 211189, People’s Republic of China

## Abstract

The crystal structure of the title compound C_8_H_10_N_3_^+^·ClO_4_^−^·C_12_H_24_O_6_·H_2_O, consists of an organic (1*H*-benzimidazol-2-yl)methanaminium cation, an inorganic ClO_4_^−^ anion, one 18-crown-6 mol­ecule and one water mol­ecule. In the crystal, the cations and 18-crown-6 mol­ecules are linked by N—H⋯O hydrogen bonds. The crystal packing is stabilized by inter­molecular O—H⋯O, O—H⋯N and O—H⋯Cl hydrogen bonds between anions and the water mol­ecules. One 18-crown-6 C atom and a perchlorate O atom are disordered; both have an occupancy factor ratio of 0.60 (2) and 0.40 (2).

## Related literature

The title compound was studied during efforts to obtain potential ferroelectric phase-transition materials. For general background to ferroelectric metal-organic frameworks, see: Fu *et al.* (2009 ▶); Ye *et al.* (2006 ▶); Zhang *et al.* (2008 ▶, 2010 ▶).
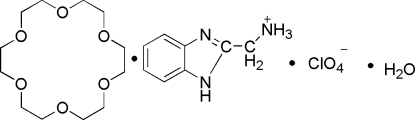


## Experimental

### 

#### Crystal data

C_8_H_10_N_3_^+^·ClO_4_^−^·C_12_H_24_O_6_·H_2_O
*M* *_r_* = 529.97
Monoclinic, 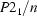

*a* = 11.703 (2) Å
*b* = 18.623 (4) Å
*c* = 12.477 (3) Å
β = 106.06 (3)°
*V* = 2613.2 (9) Å^3^
*Z* = 4
Mo *K*α radiation
μ = 0.21 mm^−1^
*T* = 293 K
0.20 × 0.20 × 0.20 mm

#### Data collection

Rigaku Mercury2 diffractometer
Absorption correction: multi-scan (*CrystalClear*; Rigaku, 2005 ▶) *T*_min_ = 0.161, *T*_max_ = 0.183
26941 measured reflections
5984 independent reflections
2947 reflections with *I* > 2σ(*I*)
*R*_int_ = 0.097

#### Refinement

*R*[*F* ^2^ > 2σ(*F* ^2^)] = 0.076
*wR*(*F* ^2^) = 0.236
*S* = 1.04
5984 reflections
343 parameters
H atoms treated by a mixture of independent and constrained refinement
Δρ_max_ = 0.49 e Å^−3^
Δρ_min_ = −0.48 e Å^−3^

### 

Data collection: *CrystalClear* (Rigaku, 2005 ▶); cell refinement: *CrystalClear*; data reduction: *CrystalClear*; program(s) used to solve structure: *SHELXS97* (Sheldrick, 2008 ▶); program(s) used to refine structure: *SHELXL97* (Sheldrick, 2008 ▶); molecular graphics: *SHELXTL* (Sheldrick, 2008 ▶); software used to prepare material for publication: *SHELXTL*.

## Supplementary Material

Crystal structure: contains datablock(s) I, global. DOI: 10.1107/S1600536811050665/bx2375sup1.cif

Structure factors: contains datablock(s) I. DOI: 10.1107/S1600536811050665/bx2375Isup2.hkl

Supplementary material file. DOI: 10.1107/S1600536811050665/bx2375Isup3.cml

Additional supplementary materials:  crystallographic information; 3D view; checkCIF report

## Figures and Tables

**Table 1:** Hydrogen-bond geometry (Å, °)

*D*—H⋯*A*	*D*—H	H⋯*A*	*D*⋯*A*	*D*—H⋯*A*
N2—H2*C*⋯O10	0.90	2.26	3.032 (6)	143
N2—H2*C*⋯O9	0.90	2.45	3.318 (7)	162
N3—H3*A*⋯O1	0.90	2.10	2.850 (4)	141
N3—H3*A*⋯O2	0.90	2.31	2.940 (4)	127
N3—H3*B*⋯O3	0.90	2.06	2.831 (4)	143
N3—H3*B*⋯O4	0.90	2.24	2.965 (4)	137
N3—H3*C*⋯O5	0.90	2.12	2.934 (4)	151
N3—H3*C*⋯O6	0.90	2.39	2.964 (4)	122
O1*W*—H1*WA*⋯N1	0.90 (7)	2.01 (7)	2.873 (5)	161 (6)
O1*W*—H1*WB*⋯O7^i^	0.78 (6)	2.50 (6)	3.202 (6)	150 (6)
O1*W*—H1*WB*⋯Cl1^i^	0.78 (6)	2.98 (6)	3.758 (5)	173 (6)

Symmetry code: (i) 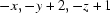.

**Fig. 1.**
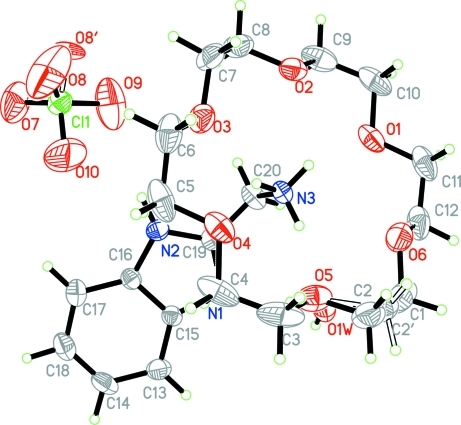
The molecular structure of the title compound, with the atomic numbering scheme. The O8 and C2 atoms are disordered. Displacement ellipsoids are drawn at the 30% probability level.

**Fig. 2.**
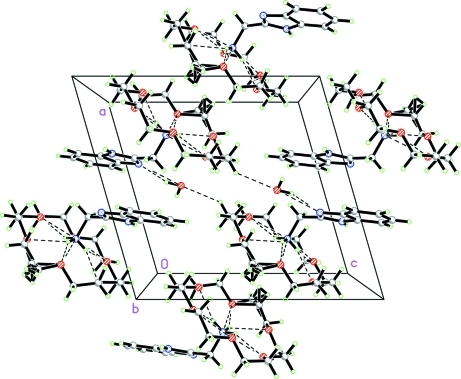
Packing diagram.

## supplementary crystallographic information

### Comment

The study of ferroelectric materials has received much attention and some materials have predominantly dielectric–ferroelectric performance (Ye *et al.*, 2006; Fu *et al.*, 2009; Zhang *et al.* 2010; Zhang *et al.*, 2008). As a part of our work to obtain potential ferroelectric phase-transition materials, we report here the crystal structure of title compound. Unluckily, the title compound has no dielectric anomalies in the temperature range 93–453 K, suggesting that it might be only a paraelectric.

The asymmetric unit of the title compoundis is shown in Fig. 1.The structure of the title compound *C*_8_H_10_N_3_^+^.ClO_4_^- ^.C_12_H_24_O_6_.H_2_O, consists of an organic 2-(1*H*-benzimidazol-2-yl)-ethylammonium cation, and inorganic ion (ClO_4_)^-^ one 18-crown-6 molecule and one water molecule. In the asymmetric unit the cations and the 18-crown-6 molecules are linked by by N—H···O hydrogen bonds. The crystal packing is stabilized by intermolecular O—H···O and O—H···Cl hydrogen bonds between anions and water molecules, Table 1. The C2 and O8 atoms are disordered with ocupation factors of 0.60 (2)/0.40 (2) respectively.

### Experimental

2-(1*H*-benzimidazol-2-yl)-ethylamine (0.09 g) and an excess of perchloric acid (0.302 g) were dissolved in methanol. Then, 18-crown-6 (0.528 g) was added to the mixture. The precipitate was filtered and washed with a small amount of methanol. Single crystals suitable for X-ray diffraction were obtained by slow evaporation of a methanol solution at room temperature over two days.

### Refinement

H atoms were placed in calculated positions (N—H = 0.90 Å and C—H = 0.97 Å for C*sp^2^* atoms), assigned fixed *U*_iso_ values [*U*_iso_ = 1.2*U*_eq_(C*sp^2^*) and 1.5*U*_eq_( *N*)] and allowed to ride. The H1WA and H1WB on the O1W were refined freely along their isotropic displacement parameters. The disordered atoms O8 and C2 were split over two sites, the occupancies of which were refined with anisotropics models to a final occupancy of 0.60 (2)/0.40 (2).

### Crystal data

**Table:**

C_8_H_10_N_3_^+^·ClO_4_^−^·C_12_H_24_O_6_·H_2_O	*Z* = 4
*M**_r_* = 529.97	*F*(000) = 1128
Monoclinic, *P*2_1_/*n*	*D*_x_ = 1.347 Mg m^−^^3^
Hall symbol: -P 2yn	Mo *K*α radiation, λ = 0.71073 Å
*a* = 11.703 (2) Å	θ = 3.0–27.5°
*b* = 18.623 (4) Å	µ = 0.21 mm^−^^1^
*c* = 12.477 (3) Å	*T* = 293 K
β = 106.06 (3)°	Prism, colourless
*V* = 2613.2 (9) Å^3^	0.20 × 0.20 × 0.20 mm

### Data collection

**Table:**

Rigaku Mercury2 diffractometer	5984 independent reflections
Radiation source: fine-focus sealed tube	2947 reflections with *I* > 2σ(*I*)
graphite	*R*_int_ = 0.097
Detector resolution: 13.6612 pixels mm^-1^	θ_max_ = 27.5°, θ_min_ = 3.0°
CCD_Profile_fitting scans	*h* = −15→15
Absorption correction: multi-scan (*CrystalClear*; Rigaku, 2005)	*k* = −24→24
*T*_min_ = 0.161, *T*_max_ = 0.183	*l* = −16→16
26941 measured reflections	

### Refinement

**Table:**

Refinement on *F*^2^	Primary atom site location: structure-invariant direct methods
Least-squares matrix: full	Secondary atom site location: difference Fourier map
*R*[*F*^2^ > 2σ(*F*^2^)] = 0.076	Hydrogen site location: inferred from neighbouring sites
*wR*(*F*^2^) = 0.236	H atoms treated by a mixture of independent and constrained refinement
*S* = 1.04	*w* = 1/[σ^2^(*F*_o_^2^) + (0.0924*P*)^2^ + 1.3318*P*] where *P* = (*F*_o_^2^ + 2*F*_c_^2^)/3
5984 reflections	(Δ/σ)_max_ = 0.001
343 parameters	Δρ_max_ = 0.49 e Å^−^^3^
0 restraints	Δρ_min_ = −0.48 e Å^−^^3^

### Special details

**Table:**

Geometry. All e.s.d.'s (except the e.s.d. in the dihedral angle between two l.s. planes) are estimated using the full covariance matrix. The cell e.s.d.'s are taken into account individually in the estimation of e.s.d.'s in distances, angles and torsion angles; correlations between e.s.d.'s in cell parameters are only used when they are defined by crystal symmetry. An approximate (isotropic) treatment of cell e.s.d.'s is used for estimating e.s.d.'s involving l.s. planes.
Refinement. Refinement of *F*^2^ against ALL reflections. The weighted *R*-factor *wR* and goodness of fit *S* are based on *F*^2^, conventional *R*-factors *R* are based on *F*, with *F* set to zero for negative *F*^2^. The threshold expression of *F*^2^ > σ(*F*^2^) is used only for calculating *R*-factors(gt) *etc*. and is not relevant to the choice of reflections for refinement. *R*-factors based on *F*^2^ are statistically about twice as large as those based on *F*, and *R*- factors based on ALL data will be even larger.

### Fractional atomic coordinates and isotropic or equivalent isotropic displacement parameters (Å^2^)

**Table:**

	*x*	*y*	*z*	*U*_iso_*/*U*_eq_	Occ. (<1)
Cl1	0.26044 (10)	0.79963 (5)	0.37379 (9)	0.0645 (3)	
O7	0.2014 (4)	0.7892 (3)	0.2618 (3)	0.1347 (16)	
O9	0.1810 (5)	0.8015 (3)	0.4416 (4)	0.175 (2)	
O10	0.2964 (6)	0.8694 (2)	0.3903 (5)	0.190 (3)	
O3	0.3959 (2)	0.84535 (16)	0.6880 (2)	0.0724 (8)	
O1	0.1554 (2)	0.87756 (16)	0.9402 (2)	0.0740 (8)	
O2	0.2686 (2)	0.78555 (13)	0.8289 (2)	0.0663 (8)	
O4	0.4890 (2)	0.98277 (17)	0.7326 (3)	0.0785 (9)	
O5	0.3883 (3)	1.07751 (15)	0.8618 (3)	0.0820 (9)	
O6	0.2657 (3)	1.01143 (17)	1.0000 (2)	0.0822 (9)	
C10	0.1543 (4)	0.8026 (3)	0.9562 (5)	0.0923 (16)	
H10A	0.0821	0.7887	0.9746	0.111*	
H10B	0.2217	0.7886	1.0174	0.111*	
C5	0.4954 (4)	0.9450 (3)	0.6367 (4)	0.0954 (17)	
H5A	0.5649	0.9601	0.6146	0.114*	
H5B	0.4253	0.9549	0.5756	0.114*	
C8	0.2836 (5)	0.7504 (2)	0.7331 (4)	0.0878 (15)	
H8A	0.2192	0.7634	0.6685	0.105*	
H8B	0.2814	0.6988	0.7430	0.105*	
C1	0.2837 (7)	1.0867 (3)	0.9978 (5)	0.1062 (19)	
H1A	0.2893	1.1070	1.0707	0.127*	
H1B	0.2168	1.1089	0.9442	0.127*	
C6	0.5029 (4)	0.8666 (3)	0.6623 (4)	0.0907 (15)	
H6A	0.5120	0.8397	0.5986	0.109*	
H6B	0.5711	0.8569	0.7253	0.109*	
C9	0.1603 (4)	0.7663 (3)	0.8530 (5)	0.0906 (16)	
H9A	0.1572	0.7147	0.8622	0.109*	
H9B	0.0931	0.7804	0.7917	0.109*	
C12	0.1522 (5)	0.9938 (3)	1.0155 (4)	0.0958 (17)	
H12A	0.0894	1.0064	0.9493	0.115*	
H12B	0.1398	1.0206	1.0781	0.115*	
C7	0.3976 (5)	0.7710 (2)	0.7148 (4)	0.0879 (15)	
H7A	0.4615	0.7614	0.7816	0.105*	
H7B	0.4117	0.7428	0.6544	0.105*	
C11	0.1492 (5)	0.9165 (3)	1.0371 (4)	0.0956 (18)	
H11A	0.2157	0.9034	1.0999	0.115*	
H11B	0.0762	0.9044	1.0556	0.115*	
C3	0.4937 (5)	1.0921 (3)	0.8285 (6)	0.107 (2)	
H3D	0.5626	1.0731	0.8836	0.129*	
H3E	0.5039	1.1435	0.8233	0.129*	
C2	0.410 (3)	1.1034 (16)	0.963 (2)	0.094 (7)	0.60 (2)
H2A	0.4256	1.1545	0.9633	0.112*	0.60 (2)
H2B	0.4772	1.0795	1.0129	0.112*	0.60 (2)
O8	0.3692 (12)	0.7681 (13)	0.4020 (12)	0.198 (9)	0.60 (2)
O8'	0.302 (3)	0.7372 (6)	0.4164 (14)	0.156 (9)	0.40 (2)
C2'	0.382 (3)	1.098 (2)	0.988 (3)	0.061 (6)	0.40 (2)
H2'A	0.4393	1.0695	1.0425	0.073*	0.40 (2)
H2'B	0.4019	1.1483	1.0034	0.073*	0.40 (2)
C4	0.4830 (5)	1.0583 (3)	0.7191 (6)	0.1035 (19)	
H4A	0.4079	1.0716	0.6668	0.124*	
H4B	0.5469	1.0747	0.6895	0.124*	
N1	0.1256 (2)	1.05970 (15)	0.6110 (2)	0.0482 (7)	
N2	0.1753 (2)	0.97320 (15)	0.5110 (2)	0.0485 (7)	
H2C	0.1942	0.9290	0.4923	0.058*	
C16	0.1647 (3)	1.03409 (18)	0.4465 (3)	0.0441 (8)	
C19	0.1515 (3)	0.99175 (18)	0.6072 (3)	0.0433 (8)	
C17	0.1780 (3)	1.0462 (2)	0.3411 (3)	0.0608 (10)	
H17A	0.1975	1.0081	0.2974	0.073*	
C20	0.1529 (3)	0.9401 (2)	0.6987 (3)	0.0568 (10)	
H20A	0.1388	0.8925	0.6681	0.068*	
H20B	0.0895	0.9519	0.7307	0.068*	
C18	0.1608 (4)	1.1156 (3)	0.3020 (3)	0.0676 (11)	
H18A	0.1701	1.1265	0.2297	0.081*	
C15	0.1332 (3)	1.08788 (17)	0.5099 (3)	0.0442 (8)	
C13	0.1150 (4)	1.15770 (19)	0.4680 (3)	0.0615 (10)	
H13A	0.0942	1.1959	0.5107	0.074*	
C14	0.1297 (4)	1.1696 (2)	0.3631 (3)	0.0666 (11)	
H14A	0.1178	1.2171	0.3320	0.080*	
N3	0.2672 (2)	0.94099 (15)	0.7874 (2)	0.0471 (7)	
H3A	0.2650	0.9093	0.8412	0.071*	
H3B	0.3264	0.9292	0.7576	0.071*	
H3C	0.2798	0.9854	0.8167	0.071*	
O1W	0.0112 (4)	1.0968 (2)	0.7796 (3)	0.0817 (10)	
H1WA	0.029 (6)	1.084 (3)	0.717 (6)	0.15 (3)*	
H1WB	−0.042 (6)	1.121 (3)	0.745 (5)	0.13 (3)*	

### Atomic displacement parameters (Å^2^)

**Table:**

	*U*^11^	*U*^22^	*U*^33^	*U*^12^	*U*^13^	*U*^23^
Cl1	0.0740 (7)	0.0562 (6)	0.0617 (6)	0.0005 (5)	0.0158 (5)	−0.0046 (5)
O7	0.145 (4)	0.177 (4)	0.069 (2)	0.017 (3)	0.008 (2)	−0.031 (2)
O9	0.139 (4)	0.287 (7)	0.117 (4)	−0.026 (4)	0.065 (3)	0.007 (4)
O10	0.285 (7)	0.094 (3)	0.218 (6)	−0.065 (4)	0.114 (5)	−0.044 (3)
O3	0.0712 (18)	0.080 (2)	0.0722 (19)	0.0159 (15)	0.0301 (15)	−0.0040 (15)
O1	0.0677 (18)	0.087 (2)	0.0759 (19)	0.0182 (15)	0.0343 (15)	0.0373 (16)
O2	0.0725 (18)	0.0500 (15)	0.0728 (18)	−0.0037 (13)	0.0139 (15)	0.0061 (13)
O4	0.0725 (19)	0.087 (2)	0.081 (2)	−0.0020 (16)	0.0295 (16)	0.0277 (17)
O5	0.067 (2)	0.0606 (18)	0.099 (3)	−0.0068 (15)	−0.0086 (18)	−0.0038 (17)
O6	0.090 (2)	0.082 (2)	0.0666 (19)	0.0269 (17)	0.0089 (17)	−0.0031 (16)
C10	0.074 (3)	0.097 (4)	0.114 (4)	0.003 (3)	0.040 (3)	0.056 (3)
C5	0.057 (3)	0.165 (5)	0.073 (3)	0.005 (3)	0.033 (2)	0.035 (4)
C8	0.120 (4)	0.052 (3)	0.079 (3)	0.003 (3)	0.007 (3)	−0.009 (2)
C1	0.125 (5)	0.093 (4)	0.082 (4)	0.027 (4)	−0.002 (4)	−0.029 (3)
C6	0.067 (3)	0.140 (5)	0.071 (3)	0.022 (3)	0.027 (2)	−0.009 (3)
C9	0.069 (3)	0.073 (3)	0.123 (4)	−0.017 (2)	0.015 (3)	0.029 (3)
C12	0.107 (4)	0.127 (5)	0.062 (3)	0.054 (3)	0.037 (3)	0.011 (3)
C7	0.114 (4)	0.071 (3)	0.080 (3)	0.034 (3)	0.029 (3)	−0.013 (3)
C11	0.090 (3)	0.139 (5)	0.074 (3)	0.049 (3)	0.050 (3)	0.042 (3)
C3	0.073 (3)	0.059 (3)	0.165 (6)	−0.019 (2)	−0.008 (4)	0.024 (3)
C2	0.116 (16)	0.061 (7)	0.089 (14)	−0.010 (9)	0.004 (9)	−0.011 (8)
O8	0.117 (9)	0.286 (18)	0.149 (10)	0.142 (10)	−0.031 (6)	−0.060 (10)
O8'	0.32 (3)	0.049 (7)	0.130 (10)	0.068 (9)	0.106 (14)	0.051 (6)
C2'	0.063 (11)	0.058 (11)	0.051 (11)	−0.002 (9)	−0.003 (9)	−0.014 (8)
C4	0.078 (3)	0.091 (4)	0.144 (5)	−0.014 (3)	0.033 (4)	0.054 (4)
N1	0.0532 (17)	0.0501 (17)	0.0412 (16)	0.0046 (14)	0.0129 (13)	0.0023 (13)
N2	0.0451 (16)	0.0456 (16)	0.0541 (17)	0.0036 (13)	0.0123 (13)	−0.0012 (14)
C16	0.0408 (18)	0.0480 (19)	0.0429 (18)	−0.0026 (15)	0.0107 (15)	0.0038 (16)
C19	0.0332 (16)	0.053 (2)	0.0415 (18)	0.0006 (14)	0.0064 (14)	0.0048 (15)
C17	0.057 (2)	0.079 (3)	0.050 (2)	−0.002 (2)	0.0196 (18)	−0.004 (2)
C20	0.0402 (19)	0.071 (2)	0.055 (2)	−0.0083 (17)	0.0049 (17)	0.0164 (19)
C18	0.069 (3)	0.086 (3)	0.046 (2)	−0.019 (2)	0.013 (2)	0.008 (2)
C15	0.0440 (18)	0.0446 (19)	0.0415 (18)	−0.0007 (15)	0.0077 (15)	0.0024 (15)
C13	0.074 (3)	0.046 (2)	0.056 (2)	0.0006 (19)	0.004 (2)	−0.0010 (18)
C14	0.074 (3)	0.059 (2)	0.056 (2)	−0.016 (2)	−0.001 (2)	0.020 (2)
N3	0.0501 (16)	0.0506 (16)	0.0407 (15)	−0.0012 (13)	0.0130 (13)	0.0069 (13)
O1W	0.100 (3)	0.086 (2)	0.066 (2)	0.017 (2)	0.035 (2)	0.0094 (18)

### Geometric parameters (Å, °)

**Table:**

Cl1—O8'	1.315 (12)	C11—H11A	0.9699
Cl1—O8	1.357 (7)	C11—H11B	0.9700
Cl1—O10	1.364 (4)	C3—C4	1.477 (8)
Cl1—O7	1.390 (4)	C3—H3D	0.9700
Cl1—O9	1.420 (5)	C3—H3E	0.9701
O3—C7	1.424 (5)	C2—H2A	0.9700
O3—C6	1.431 (5)	C2—H2B	0.9699
O1—C10	1.411 (5)	C2—H2'A	1.1500
O1—C11	1.429 (6)	C2—H2'B	0.9971
O2—C8	1.415 (5)	C2'—H2A	1.2461
O2—C9	1.427 (5)	C2'—H2B	1.1259
O4—C5	1.409 (6)	C2'—H2'A	0.9699
O4—C4	1.416 (5)	C2'—H2'B	0.9700
O5—C2	1.30 (2)	C4—H4A	0.9700
O5—C3	1.432 (7)	C4—H4B	0.9701
O5—C2'	1.65 (3)	N1—C19	1.305 (4)
O6—C1	1.420 (6)	N1—C15	1.391 (4)
O6—C12	1.434 (6)	N2—C19	1.350 (4)
C10—C9	1.474 (7)	N2—C16	1.376 (4)
C10—H10A	0.9701	N2—H2C	0.9001
C10—H10B	0.9699	C16—C17	1.385 (5)
C5—C6	1.492 (7)	C16—C15	1.389 (4)
C5—H5A	0.9700	C19—C20	1.490 (5)
C5—H5B	0.9700	C17—C18	1.376 (6)
C8—C7	1.465 (7)	C17—H17A	0.9599
C8—H8A	0.9700	C20—N3	1.482 (4)
C8—H8B	0.9700	C20—H20A	0.9599
C1—C2'	1.21 (3)	C20—H20B	0.9599
C1—C2	1.68 (3)	C18—C14	1.370 (6)
C1—H1A	0.9699	C18—H18A	0.9600
C1—H1B	0.9699	C15—C13	1.396 (5)
C6—H6A	0.9699	C13—C14	1.384 (5)
C6—H6B	0.9700	C13—H13A	0.9599
C9—H9A	0.9701	C14—H14A	0.9601
C9—H9B	0.9700	N3—H3A	0.9000
C12—C11	1.467 (7)	N3—H3B	0.9000
C12—H12A	0.9699	N3—H3C	0.9000
C12—H12B	0.9700	O1W—H1WA	0.90 (7)
C7—H7A	0.9700	O1W—H1WB	0.78 (6)
C7—H7B	0.9700		
			
O8'—Cl1—O8	45.2 (8)	O5—C3—H3D	109.9
O8'—Cl1—O10	135.7 (13)	C4—C3—H3D	109.9
O8—Cl1—O10	98.3 (11)	O5—C3—H3E	109.8
O8'—Cl1—O7	108.0 (9)	C4—C3—H3E	109.8
O8—Cl1—O7	111.7 (5)	H3D—C3—H3E	108.3
O10—Cl1—O7	109.4 (3)	O5—C2—C1	103.6 (18)
O8'—Cl1—O9	90.8 (10)	O5—C2—H2A	111.0
O8—Cl1—O9	125.4 (10)	C1—C2—H2A	110.9
O10—Cl1—O9	96.4 (4)	O5—C2—H2B	111.1
O7—Cl1—O9	112.1 (3)	C1—C2—H2B	111.2
C7—O3—C6	111.4 (4)	H2A—C2—H2B	109.0
C10—O1—C11	112.2 (4)	O5—C2—H2'A	124.4
C8—O2—C9	112.7 (4)	C1—C2—H2'A	75.3
C5—O4—C4	114.0 (4)	H2A—C2—H2'A	121.4
C2—O5—C3	104.9 (13)	O5—C2—H2'B	141.5
C3—O5—C2'	120.4 (11)	C1—C2—H2'B	79.0
C1—O6—C12	112.1 (4)	H2B—C2—H2'B	103.1
O1—C10—C9	109.0 (4)	H2'A—C2—H2'B	93.7
O1—C10—H10A	109.9	C1—C2'—O5	111 (2)
C9—C10—H10A	109.9	C1—C2'—H2A	129.8
O1—C10—H10B	109.9	O5—C2'—H2A	79.9
C9—C10—H10B	109.9	C1—C2'—H2B	144.9
H10A—C10—H10B	108.3	O5—C2'—H2B	83.4
O4—C5—C6	108.7 (4)	H2A—C2'—H2B	83.3
O4—C5—H5A	109.9	C1—C2'—H2'A	109.3
C6—C5—H5A	109.9	O5—C2'—H2'A	109.5
O4—C5—H5B	109.9	H2A—C2'—H2'A	112.6
C6—C5—H5B	110.0	H2B—C2'—H2'A	36.7
H5A—C5—H5B	108.3	C1—C2'—H2'B	109.6
O2—C8—C7	110.0 (4)	O5—C2'—H2'B	109.7
O2—C8—H8A	109.7	H2B—C2'—H2'B	94.3
C7—C8—H8A	109.6	H2'A—C2'—H2'B	108.1
O2—C8—H8B	109.7	O4—C4—C3	108.9 (4)
C7—C8—H8B	109.7	O4—C4—H4A	109.9
H8A—C8—H8B	108.2	C3—C4—H4A	110.0
C2'—C1—O6	109.0 (18)	O4—C4—H4B	109.9
C2'—C1—C2	9(2)	C3—C4—H4B	109.9
O6—C1—C2	109.4 (11)	H4A—C4—H4B	108.3
C2'—C1—H1A	101.8	C19—N1—C15	105.0 (3)
O6—C1—H1A	109.8	C19—N2—C16	107.6 (3)
C2—C1—H1A	109.7	C19—N2—H2C	126.2
C2'—C1—H1B	117.9	C16—N2—H2C	126.3
O6—C1—H1B	109.8	N2—C16—C17	132.4 (3)
C2—C1—H1B	110.0	N2—C16—C15	104.9 (3)
H1A—C1—H1B	108.2	C17—C16—C15	122.7 (3)
O3—C6—C5	108.4 (4)	N1—C19—N2	112.8 (3)
O3—C6—H6A	110.0	N1—C19—C20	123.5 (3)
C5—C6—H6A	110.0	N2—C19—C20	123.7 (3)
O3—C6—H6B	110.0	C18—C17—C16	116.5 (4)
C5—C6—H6B	110.1	C18—C17—H17A	121.8
H6A—C6—H6B	108.4	C16—C17—H17A	121.7
O2—C9—C10	109.2 (4)	N3—C20—C19	112.4 (3)
O2—C9—H9A	109.8	N3—C20—H20A	109.1
C10—C9—H9A	109.8	C19—C20—H20A	109.1
O2—C9—H9B	109.8	N3—C20—H20B	109.1
C10—C9—H9B	109.9	C19—C20—H20B	109.0
H9A—C9—H9B	108.3	H20A—C20—H20B	108.1
O6—C12—C11	108.7 (4)	C14—C18—C17	121.8 (4)
O6—C12—H12A	110.0	C14—C18—H18A	119.0
C11—C12—H12A	110.0	C17—C18—H18A	119.1
O6—C12—H12B	109.9	C16—C15—N1	109.8 (3)
C11—C12—H12B	110.0	C16—C15—C13	119.7 (3)
H12A—C12—H12B	108.3	N1—C15—C13	130.6 (3)
O3—C7—C8	109.7 (4)	C14—C13—C15	117.3 (4)
O3—C7—H7A	109.7	C14—C13—H13A	121.6
C8—C7—H7A	109.7	C15—C13—H13A	121.1
O3—C7—H7B	109.8	C18—C14—C13	122.0 (4)
C8—C7—H7B	109.8	C18—C14—H14A	118.9
H7A—C7—H7B	108.2	C13—C14—H14A	119.1
O1—C11—C12	109.4 (4)	C20—N3—H3A	110.1
O1—C11—H11A	109.8	C20—N3—H3B	109.2
C12—C11—H11A	109.9	H3A—N3—H3B	109.5
O1—C11—H11B	109.8	C20—N3—H3C	109.1
C12—C11—H11B	109.7	H3A—N3—H3C	109.5
H11A—C11—H11B	108.2	H3B—N3—H3C	109.5
O5—C3—C4	109.1 (4)	H1WA—O1W—H1WB	91 (6)

### Hydrogen-bond geometry (Å, °)

**Table:**

*D*—H···*A*	*D*—H	H···*A*	*D*···*A*	*D*—H···*A*
N2—H2C···O10	0.90	2.26	3.032 (6)	143.
N2—H2C···O9	0.90	2.45	3.318 (7)	162.
N3—H3A···O1	0.90	2.10	2.850 (4)	141.
N3—H3A···O2	0.90	2.31	2.940 (4)	127.
N3—H3B···O3	0.90	2.06	2.831 (4)	143.
N3—H3B···O4	0.90	2.24	2.965 (4)	137.
N3—H3C···O5	0.90	2.12	2.934 (4)	151.
N3—H3C···O6	0.90	2.39	2.964 (4)	122.
O1W—H1WA···N1	0.90 (7)	2.01 (7)	2.873 (5)	161 (6)
O1W—H1WB···O7^i^	0.78 (6)	2.50 (6)	3.202 (6)	150 (6)
O1W—H1WB···Cl1^i^	0.78 (6)	2.98 (6)	3.758 (5)	173 (6)

Symmetry codes: (i) −*x*, −*y*+2, −*z*+1.
